# Ovarian Cyst, Ovariotomy, Menstruation from the Pedicle

**Published:** 1876-04

**Authors:** T. F. Prewitt

**Affiliations:** St. Louis, Mo.


### MISCELLANEOUS.

### Ovarian Cyst; four Tappings in eleven years; two Labors at Full Term, and one Miscarriage during existence of the Tumor; Ovariotomy; Recovery; Menstruation from the Pedicle.

By T. F. Prewitt, M. D., of St. Louis, Mo.

MRS. O., æt. 39, the mother of six children; first discovered the existence of a tumor in the abdomen immediately after her confinement at full term, August 14, 1864. Three months after this was tapped for the first time, and has been tapped three times since, at intervals of two or three years; four to six gallons of fluid were drawn at each tapping; the last was May, 1874. She was again delivered of a child at full term December 23, 1866, and had a miscarriage at four months October, 1874. After the drawing off of the fluid, there was always a great improvement in her health and strength for about a year; but when the fluid reaccumulated, her health again suffered, she became emaciated, œdema of the lower extremities occured, etc. In September last great distention had again occurred; she had already become considerably emaciated, and her feet had begun to swell. Made a diagnosis of ovarian tumour, a single large cyst, free from pelvic adhesions, and advised its removal. After explaining to her that tapping was not free from danger, that it was, at best, but a palliative measure, and that each resort to it exhausted her the more and diminished her chances of recovery from the only curative measure, ovariotomy, she decided, with a full knowledge of the dangers connected with it, to submit to the operation.

Every precaution was then taken to guard against septic infection. The house, fortunately, was a new one, well lighted, with a southern exposure. The bedding, clothing, towels and instruments were all thoroughly disinfected with a solution of carbolic acid, and clean, new sponges prepared.

At 12 o'clock, October 14, in the presence of Drs. J. T. Hodgen, A. P. Lankford, J. M. Scott, P. G. Robinson, J. K. Bauduy, B. M. Hypes and W. A. McCandless, to all of whom I am much indebted for assistance during the operation, she was brought under the influence of Squibbs's stronger ether, Dr. McCandless kindly taking charge of the anæsthetic. She bore this well, her pulse remaining full, soft, and scarcely accelerated above the normal. As she was slow in coming under the influence of the ether, a few drops of chloroform were poured upon the napkin. At my request Dr. Scott introduced a catheter, and emptied the bladder. An incision in the median line of two inches and a half in length was first made, and subsequently enlarged to five inches.

The tumor was found free from adhesions, and a Wells trocar and canula, with tubing attached, was thrust in, and about two buckets full of a pale straw-colored fluid drawn off.

The sac was found to be connected with the right ovary, and to have for its pedicle nearly the whole of the breadth of the broad ligament, making a very short pedicle, with a breadth of at least six inches.

The Fallopian tube could be seen running along near the base of the tumor, greatly lengthened, extending up upon the side of the cyst, with the fimbriated extremity still well marked.

I had already determined, if the case was a favorable one for it, to try enucleation, as first suggested and practiced by Prof. Miner, of Buffalo. Accordingly, having first nicked the peritoneum of the pedicle, I inserted my finger under the base of the tumor, and commenced the process of peeling off the peritoneum, with the bloodvessels, from the cyst-wall proper. In the collapsed condition of the tumor I found some difficulty in doing this. I therefore requested Dr. Hodgen to introduce his hand, and carry it to the bottom of the cyst, to serve as a point of support. This facilitated the matter somewhat, but unfortunately I ruptured a large vein running along with the Fallopian tube, which bled to such an extent as to require ligature.

This decided me to abandon further attempts at enucleation and apply a clamp. The vein was compressed at the point of rupture, and the cavity that had been occupied by the base of the tumor sponged out. It was found that there *was no bleeding from this surface,* and instead of a broad, short pedicle, I had a long narrow one, made up of the collapsed peritoneum, areolar tissue, bloodvessels, etc., that had enclosed the base of the tumor.

A Well's Clamp was applied as high as the rupture in the vein would permit, and the pedicle cut off two and a half inches above it. The pelvic cavity was now thoroughly cleansed by sponging out every drop of blood and serum, and the incison brought together around the pedicle by six silver wire sutures carried through the whole thickness of the abdominal walls, *including* the *peritoneum.* The usual compress and bandages were applied, and the patient put to bed at about one o'clock.

Had some nausea and made one or two efforts at vomiting while recovering from the anæsthetic, to be attributed perhaps to the small amount of chloroform administered with the ether. The abdomen was firmly supported during these efforts, and the nausea soon subsided. Some evidences of shock, too, manifested themselves as the anæsthesia wore off, but these soon disappeared under appropriate treatment.

A hypodermic injection of morphia, gr. ½, was administered, and the patient was very soon comfortable.

6.30 P. M. Pulse 80; of good volume; skin warm; drew of *two pints* of healthy urine, showing the free action of the kidneys so much insisted on by Mr. Spencer Wells. No food was allowed by the stomach, but nutritive enema of beef tea and milk directed to be given every four hours.

Hypodermic injections of morphia were given at intervals to relieve pain.

October 15. Allowed small quanties of milk and beef-tea by stomach; pulse 88; temperature 99° to 100° Fahr.

16th. Patient suffered during latter part of the night with pain and nausea, and vomited some. Vomiting recurred at 1 P. M., and the material ejected from the stomach *looks and smells like stercoraceous matter*.

All food by the stomach was again forbidden, and under the influence of morphia hypodermically, the application of sinapisms to to the epigastrium, the internal administration of hydrocyanic acid, champagne and ice, these alarming symptoms disappeared. During the further progress of the case scarcely an unpleasant symptom occured. Some flatulence of the bowels gave rise to considerable discomfort, relieved by the simple expedient of introducing the detached point of a syringe into the rectum and allowing it to remain, thus permitting large quanties of gas to escape.

Cystitis also developed from the previous existence of cystocele, and the failure to completely empty the bladder, with the repeated introduction of the catheter Quantities of thick ropy mucus were secreted, completely blocking the eye of the catheter and preventing the flow of urine. This disappeared promptly after washing out the bladder a few times with flaxseed mucilage.

Throughout the whole course of the convalescence, the pulse never exceeded 108, and the temperature never exceeded 101°.

A small abscess formed at the upper side of the pedicle, seemingly parietal, and caused the patient to be confined to bed longer than would have been necessary otherwise, and the highest temperatue (101° F. on 23d day) was reached during its formation.

In conclusion, I would say, that, though failing to carry out enucleation in this case, I am impressed with the feasibility and safety of it. Indeed, I am inclined to regard it as the most rational treatment of the pedicle in all cases adapted to it. While it would obviously be less readily accomplished in a simple cyst than in a semi-solid tumor, the failure in this case was due to myself, perhaps, rather that the inherent difficulty in the process, and my experience in this, I believe, would enable me to succeed better in a similar case in the future. I might too have adopted Prof. Miner's suggestion, and applied a metal ligature to the bleeding vein, completed the enucleation, and dropped the whole pedicle back into the abdomen as in any other case, but at the time thought it better to apply the clamp.

January 14, 1876. Mrs. O. called upon me to-day, looking greatly improved in health and rapidly gaining flesh.

A small red granulating tumor as large as a small cherry projects at the site of the pedicle, and from this *a discharge of blood takes place at each menstruation and continues during the whole of that period.* She is now menstruating for the third time since the operation, and this prenomenon has occured during each of these periods. Menstruation otherwise normal.—*American Journal Med. Sciences.*

